# Unveiling Viruses Associated with Gastroenteritis Using a Metagenomics Approach

**DOI:** 10.3390/v12121432

**Published:** 2020-12-13

**Authors:** Xavier Fernandez-Cassi, Sandra Martínez-Puchol, Marcelle Silva-Sales, Thais Cornejo, Rosa Bartolome, Silvia Bofill-Mas, Rosina Girones

**Affiliations:** 1 Laboratory of Virus Contaminants of Water and Food, Department of Genetics, Microbiology and Statistics, University of Barcelona, 08028 Barcelona, Spain; smartinezpuchol@ub.edu (S.M.-P.); marcelle.figueira@ufg.br (M.S.-S.); Sbofill@ub.edu (S.B.-M.); rgirones@ub.edu (R.G.); 2 Laboratory of Virology and Cell Culture, Tropical Pathology and Public Health Institute, Federal University of Goiás, Goiânia, GO 74605-050, Brazil; 3 Hospital Universitari Vall d’Hebron, Microbiology Service, 08035 Barcelona, Spain; thais.cornejo@vhir.org (T.C.); rbartolome@vhebron.net (R.B.)

**Keywords:** acute gastroenteritis, viral metagenomics, children, next-generation sequencing, norovirus, sapovirus, astrovirus, *Picornaviridae*

## Abstract

Acute infectious gastroenteritis is an important illness worldwide, especially on children, with viruses accounting for approximately 70% of the acute cases. A high number of these cases have an unknown etiological agent and the rise of next generation sequencing technologies has opened new opportunities for viral pathogen detection and discovery. Viral metagenomics in routine clinical settings has the potential to identify unexpected or novel variants of viral pathogens that cause gastroenteritis. In this study, 124 samples from acute gastroenteritis patients from 2012–2014 previously tested negative for common gastroenteritis pathogens were pooled by age and analyzed by next generation sequencing (NGS) to elucidate unidentified viral infections. The most abundant sequences detected potentially associated to acute gastroenteritis were from *Astroviridae* and *Caliciviridae* families, with the detection of norovirus GIV and sapoviruses. Lower number of contigs associated to rotaviruses were detected. As expected, other viruses that may be associated to gastroenteritis but also produce persistent infections in the gut were identified including several *Picornaviridae* members (EV, parechoviruses, cardioviruses) and adenoviruses. According to the sequencing data, astroviruses, sapoviruses and NoV GIV should be added to the list of viral pathogens screened in routine clinical analysis.

## 1. Introduction

Gastroenteritis is an important health problem around the globe affecting people at any age, but it is especially severe among children and the elderly. In 2016, diarrhea was the eighth leading cause of death among populations of all ages, causing an estimated 1,655,944 deaths and representing the fifth cause of death in children under 5 years with more than 446,000 estimated deaths [1]. While most of these deaths occur in developing countries, in developed countries acute gastroenteritis is still an important cause of death and an economic problem [2]. Clinical gastroenteritis can be caused by several infectious agents including bacteria, protozoa and viruses, the transmission of which can be foodborne or waterborne, through contaminated fomites or by person-to-person contact. Among all gastroenteritis etiological agents, viruses account for approximately 70% of acute cases in children with rotavirus (RV), norovirus (NoV), and adenovirus (AdV), accounting for the majority of cases [3,4], with an increase in astrovirus (AstV) and sapovirus (SaV) in the same age groups [5,6]. Despite these important viral agents, it has also been estimated that nearly 40% of gastroenteritis cases have an unknown etiological agent, suggesting that most could be caused by undiscovered viruses [7]. In recent years, the advent of next-generation sequencing (NGS) technologies has opened new opportunities for viral pathogen detection and discovery in the clinical setting [8]. Currently, more than 10,261 validated, complete viral genomes are available in databases (e.g., the National Center for Biotechnology Information (NCBI) RefSeq in September 2020, https://www.ncbi.nlm.nih.gov/genome/viruses/).

In the present manuscript, fecal samples from acute gastroenteritis patients who had previously tested negative for common pathogenic bacteria (*Salmonella* sp., *Campylobacter* sp., *Shigella* sp., *Yersinia* sp. and Verotoxigenic *E. coli* (VTEC).), parasites (*Giardia* sp. and *Cryptosporidium* sp.) and viruses (rotavirus, adenovirus, norovirus) using conventional routine methodologies, were analyzed by NGS to elucidate unidentified viral infections.

## 2. Materials and Methods

### 2.1. Sample Collection and Characterization

A total of 124 fecal samples from patients with acute gastroenteritis of unknown origin were collected in collaboration with Hospital Universitari Vall d’Hebron, Barcelona, Catalonia, Spain during 2012 and 2014. Previously, fecal samples were screened for the most common entero-pathogens, including *Salmonella* sp., *Campylobacter* sp., *Shigella* sp., *Yersinia* sp., and Verotoxigenic *E. coli* (VTEC), for which they tested negative. Samples were also screened for *Giardia* sp. and *Cryptosporidium* sp. if the clinical symptoms were compatible with a parasitic infection. In addition, frequent viral gastroenteritis agents for the specific age groups were tested using immunochromatographic assays for human adenoviruses (ONE STEP Adenovirus CARD TEST, Certest Biotec S.L), human rotaviruses (ONE STEP Rotavirus CARD TEST, Certest Biotec S.L) and human astroviruses (ONE STEP Astrovirus CARD TEST, Certest Biotec S.L), and conventional RT-PCR (norovirus GI and GII) [9]. No etiological agent could be identified to explain the clinical acute gastroenteritis; hence, the samples were screened using metagenomic means by pooling the samples according to age criteria in four different categories: less than 1 year (3 pools: F < 1, S < 1A and S < 1B), from 1 to 3 years (4 pools: F1-3A, S1-3A, S1-3B and S1-3C), from 3 to 10 years (1 pool: S3-10), and older than 10 years (1 pool: F > 10). For pools “F”, all samples were tested by RT-PCR for noroviruses [9]. Samples pooled under “S” criteria were only tested by RT-PCR under specific clinical request. A summary of the sample features composing each pool and the clinical tests performed is represented as Table 1.

### 2.2. Viral Concentration, Free DNA Removal, Nucleic Acid Extraction, and Library Preamplification and Preparation Using Untargeted Viral Metagenomics (UVM) and Target Enrichment Sequencing (TES)

Individual fecal samples of 0.5 g were suspended in 4.5 mL PBS 1x solution to obtain a 10% fecal suspension. Suspensions were homogenized and pooled by mixing 1 mL of each sample to construct the pools (Table 1). To enrich the viral fraction, fecal pools were centrifuged for 15 min at 3000× *g* to remove cellular debris and bacteria; the supernatant was collected and filtered using a 0.22-µm low-binding protein syringe filter (Millex-GV, 0.22 µm, PVDF, 33 mm, Gamma-Sterilized, Millipore, Massachusetts, USA). Filtered pools (500 µL) were treated with 230 units of Turbo DNAse (Ambion, Lithuania) for 1 h at 37 °C to remove free DNA prior to nucleic acid extraction. The NA (nucleic acids) present in 280 µL of DNAse-treated viral concentrates was extracted using the QIAamp^®^Viral RNA Mini Kit from QIAGEN (Qiagen, Valencia, CA, USA). NA were eluted in 60 µL and stored at −80 °C until further processing. To enable the detection of both DNA and RNA viruses, the total NAs were reverse-transcribed as previously described by Fernandez-Cassi et al. [10]. In short, SuperScript III (Life Technologies, Carlsbad, CA, USA) was used to retrotranscribe RNA to cDNA with primerA (5′-GTTTCCCAGTCACGATCNNNNNNNNN-3′). Second strand cDNA and DNA were constructed including primer sequences using Sequenase (USB/Affymetrix, Cleveland, OH, USA). PCR amplification with AmpliTaqGold (Life Technologies, Austin, Texas, USA) was performed using primerB (5′-GTTTCCCAGTCACGATC-3′); this step was performed in duplicate. After 10 min at 95 °C to activate DNA polymerase, the following PCR program was used: 25 cycles of 30 s at 94 °C, 30 s at 40 °C, and 30 s at 50 °C, with a final step of 60 s at 72 °C. The PCR products were purified and eluted in 15 µL using a Zymo DNA clean and concentrator (cat no. D4013, Zymo Research, Irvine, CA, USA) to yield sufficient DNA for the library preparation. Amplified DNA samples were quantified using Qubit 2.0 (cat no. Q32854, Life Technologies, OR, USA).

Untargeted viral metagenomics (UVM) viral libraries were constructed using a Nextera XT DNA sample preparation kit following the manufacturer’s instructions (Illumina Inc).

For the Target Enrichment Sequencing (TES), one pool (F2, 1–3 years) was reprocessed and resequenced by hybridizing the libraries with the vertebrate viral capture panel (VirCapSeq Enrichment Kit, Roche, Pleasanton, CA, USA) developed by Briese and coworkers [11] as detailed by Martínez-Puchol et al. [12]. Libraries were sequenced for three different runs using Illumina MiSeq 2 × 300 in base-pair paired-end format.

### 2.3. Bioinformatics Analysis

Bioinformatics analysis was performed using the Genome Detective platform (https://www.genomedetective.com/) [13]. For a more precise and accurate taxonomic classification, human viral contigs longer than 100 bp were queried for sequence similarity using BLASTN against the NCBI GenBank nucleotide collection database [14] with Geneious Software 11.1.5 (www.geneious.com) [15].

The species nomenclature and classification were performed according to the NCBI Taxonomy standards. For specific typing of human calicivirus, the assembled genomes of human noroviruses and sapoviruses were uploaded into the Noronet web-based service Typing tool (version 2.0) developed by RIVM using the updated classification of norovirus genogroups and genotypes [16,17]. Specific phylogenetic trees for Sapovirus were performed by aligning the complete genomes and the VP1 region against reference strains using MUSCLE [18]. Trees were constructed using tree view [19] software within Geneious (software version 11.0, https://www.geneious.com). For the *Enterovirus* genus within the *Picornaviridae* family, the same approach was performed, using the Enterovirus Genotyping Tool (version 0.1) developed by the RIVM [17]. For human adenovirus, retrieved contigs were mapped against their closest adenovirus type, and the hexon, penton, and fiber genes regions were annotated, extracted, and phylogenetically analyzed using the prototype strains suggested by the human Adenovirus Working Group (http://hadvwg.gmu.edu/). For human rotavirus analysis, a routinely binary phylogenetic analysis using the genes VP4 and VP7 was performed with the tool Rotavirus A Genotype Determination (ViPR, www.viprbrc.org) [20]. Contigs belonging to the *mamastrovirus* and *parechovirus* genus were aligned to their complete reference genomes according to the ICTV classification. In addition, specific alignments using the ORF2 region and VP1 region for mamastrovirus and parechovirus against selected reference strains were conducted [21,22].

### 2.4. RT-PCR and RT-qPCR for Specific Viral Pathogens Sequencing

Specific viral RT-qPCR to detect NoVGIV was performed using primers and conditions as previously described [9,23,24] for individual fecal samples. RT-PCR for norovirus genogroup II was used in a specific subset of samples to confirm the obtained NGS results [25]. RT-PCR-positive band products were purified using Zymo DNA clean and concentrator (Zymo Research, cat no. D4013, Zymo Research, Irvine, CA, USA) and submitted to Sanger sequencing at Serveis Científico-Tècnics of the University of Barcelona.

## 3. Results and Discussion

### 3.1. Metagenomic Identification of Viral Families in Pooled Fecal Samples

A summary of the MiSeq output obtained from each sequenced pool containing the raw read number, contigs, and viral sequences detected, among other information, is presented as Supplementary material 1. Approximately 44 million raw-paired ends (0.9 to 12 M per sample) were generated. On average, 95% of the reads passed the QC cut-off and were annotated as viral using the Diamond tool [26], with 46% of these reads identified as viral (23% to 88%). When analyzing the samples according to age, a minimum and maximum percentage of viral reads ranging from to 29% to 50%, 23% to 56%, and 41% to 88% were obtained for pools with individuals aged younger than 1 year (F < 1, S < 1A and S < 1B), 1 to 3 years (F1-3A, S1-3A, S1-3B and S1-3C), and 3 to 10 years (F3-10 and S-3-10), respectively. Despite efforts to enhance viral content using filtration and nuclease treatment, 54% of the reads were classified as nonviral (12–77%). In the pool enriched by viral probe capture prior to sequencing (F1-3A), 74% of the reads were classified as viral.

**Figure 1:**
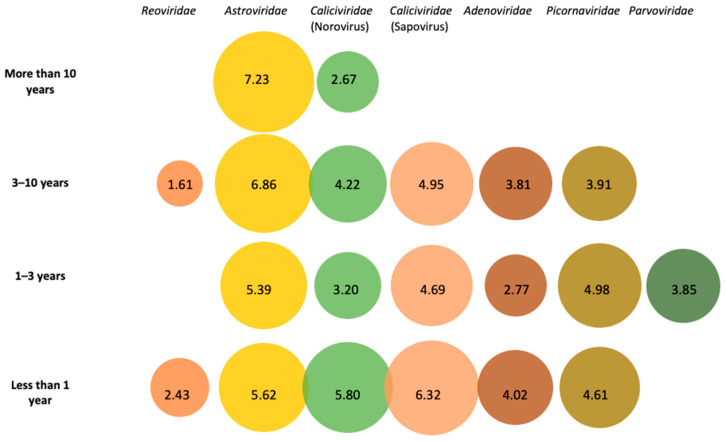
Age distribution of the number of reads (log10) from human viral families with etiologically related members with viral gastroenteritis.

A complete inventory of identified viral species classified according to the genome detective pipeline are presented in Supplementary material 2. Relevant taxonomical assignments for acute gastroenteritis will be discussed further. Additionally, these relevant pathogens which were considered important viral pathogens associated with gastroenteritis were plotted according to age and log10 abundance of reads (Figure 1).

Astrovirus, Sapovirus and NoV GIV were detected with a high number of contigs, retrieving almost their complete genomes, and are considered the potential causative agents of gastroenteritis.

### 3.2. Caliciviridae

The results for this family have been divided into the main two human genera: *Norovirus* (NoV) and *Sapovirus* (SaV).

A summary of the viral contigs identified in the pools belonging to *Caliciviridae* family is presented in Table 2.

#### 3.2.1. Norovirus

Norovirus is the leading viral agent related to acute gastroenteritis in all age groups worldwide, with frequent outbreaks related to schools, hospitals, or cruise ships [27]. Its classification into genogroups and P-types (polymerase types) is sustained using a binary system based on the amino acid diversity of the complete VP1 gene and nucleotide diversity of the RNA-dependent RNA polymerase (RdRp) [16,17]. However, due their continuous recombination, NoV taxonomical classification is constantly evolving [16]. Four main genogroups are known to infect humans: GI, GII, GIV, and GIX (formerly GII.15) [16]. Norovirus GII genogroups are responsible for the majority of acute gastroenteritis cases, with GII.4 being the most predominant genotype worldwide with different variants emerging frequently [28].

In pools S1-3A, F1-3A, and F1-3B, three NoV GII contigs were identified. Two contigs of 5978 bp and 6808 bp were classified as GII.P31-GII.2 and GII.P17-GII.17, respectively. Infections caused by GII.P17-GII.17 have been increasing over the last decade, being predominant in some Asian regions, where they have displaced the classical GII.P4-GII.4 and its recombinant strains [29]. Its presence has increased and expanded in subsequent years after the first reported case, presenting a worldwide distribution and producing outbreaks elsewhere [30,31]. According to Van Beek et al., NoV GIIP.17 and GII.17 strains were detected in most European Countries in 2015–2016, but not in Spain [29]. The detection in 2014 samples suggests that the virus was already circulating in the population outside China. To verify this finding, conventional RT-PCR was conducted by following the suggested typing protocol described by van Beek and collaborators [25]. One sample from the S1-3B pool tested positive for NoV GII, and the amplicon was submitted to the RIVM Norovirus Genotyping Tool to confirm the finding of GII.17 (Accession number: MW205840).

Finally, one contig from pool F3-10 was subtyped as NoV GII.P4-GII.4 (New Orleans 2009). As stated previously, this is the predominant genotype worldwide and accounts for more than 70% of norovirus outbreaks [32], with GII.4 subvariants emerging, cocirculating, and replacing each other over time [33,34]. This particular strain could have been considered as non-detected by conventional RT-PCR due to a failure of the assay.

In pool S < 1A, one contig of 7440 bp of NoV GII.P21-GII.3 was obtained. This recombinant strain is one of the most common strains circulating during the period under study [25,35,36]. In the same pool, one NoV GI.2-P1.2 was detected. This genotype has been linked to the consumption of polluted shellfish [37,38,39]. The non-detection of this particular genotype has been reported previously on some commercially available kits [40,41], and in-silico analysis revealed three mismatches with two of the forward primers used in clinical routine detection.

**Figure 2:**
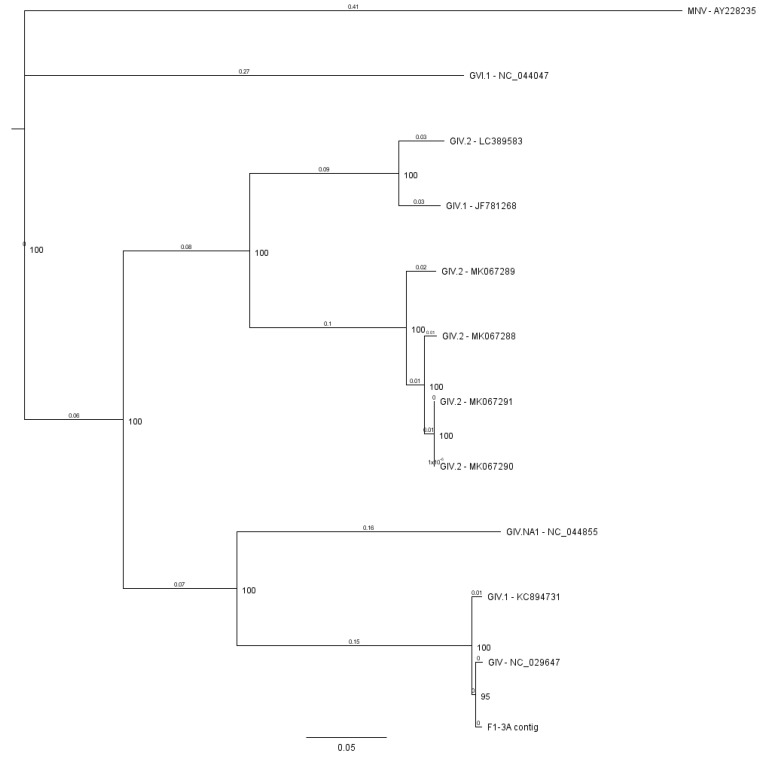
Phylogenetic tree of the almost-complete NoV GIV sequence from the F1-3A pool. Full genome sequences of NoV GIV are available at GenBank and were aligned using MUSCLE (Geneious 11.1). The murine norovirus sequence (accession number: AY228235) was used to root the tree. The tree was constructed with the Tamura-Nei genetic distance model using the neighbor-joining method with 1000 bootstrap replicates. The nodes labels indicate the consensus support whereas the labels on the branch indicate the number of substitutions per site.

Several contigs taxonomically classified as NoV GIV were retrieved from the F1-3A and F > 10 pools. To explore the real incidence of norovirus GIV, a specific RT-qPCR assay for NoV GIV was performed using individual patient samples to confirm the finding. Three of 26 samples (12% of incidence) within the affected pools were positive by the RT-qPCR assay, with viral loads ranging from 2.71 × 10^6^ to 1.34 × 10^7^ genomic copies/gr feces. The viral loads are compatible with an active replication of the virus and could explain the acute gastroenteritis of these three patients, despite its lower incidence compared with NoVGII and GI. The detected contigs could not be typed using the Noronet Typing tool. In an attempt to obtain a more precise taxonomic classification, full NoV GIV retrieved was aligned against NoV GIV full complete genomes available at genbank using MUSCLE, and a phylogenetic tree was constructed (Figure 2). Full sequenced NoV GIV shares a homology of 99.2% with the NoV GIV strain from Australia (JQ613567) and could be classified as GIV.1. Norovirus GIV was first reported in 2000 by analyzing sporadic gastroenteritis cases [42], after which two different genotypes were identified, with NoV GIV.1 affecting humans and NoV GIV.2 considered a zoonotic genotype producing gastroenteritis in animals. Norovirus infections caused by GIV.1 are rare, and its prevalence in the population remains unknown. An international molecular surveillance NoV study conducted between 2005 and 2016 showed that less than 0.1% of the sequences submitted to NoroNet belonged to the NoV GIV.1 genotype [29]. Our findings are in line with previously reported results showing an incidence of 16% of NoV GIV in clinical samples [43]. The presence of this genotype has been detected by metagenomics in samples from children affected by gastroenteritis [44], as well as in other clinical and urban wastewater samples [43,45,46], indicating that it is actively circulating in the population but probably underreported. While the introduction of routine molecular detection in clinical settings could be the key for better monitoring of GIV-related cases, the NGS techniques have been shown to be a useful tool to detect a broader spectrum of norovirus genotypes and genogroups. This is especially relevant considering that their detection by common RT-PCR can sometimes fail due to the high variability in the P1 and VP1 regions, once again lending importance to the improvement of primer-independent detection techniques [47].

#### 3.2.2. Sapovirus

Sapoviruses are one of the leading viral causes of gastroenteritis in children, with a particular impact in developing countries [48,49]. Their taxonomic classification is based on the capsid (VP1) sequence, with GI, GII, GIV and GV being the genogroups responsible for human gastroenteritis. Additionally, genogroups are further classified into genotypes, a classification system that keeps growing due to their high genetic diversity, with GI.1, GII.1, and GI.2 being the genotypes most commonly associated with clinical cases [48,50,51].

Contigs from the *sapovirus* genus were found in all sample pools except F > 10 (Table 2). Most of the detected sapoviruses were classified as GI (11/14), the most prevalent genogroup in patients within the studied age range (1 to 10 years) [49]. The typed GI contigs were GI.2 and GI.3, but no contig belonging to GI.1 was identified despite being the most prevalent in gastroenteritis samples and in this patient age group [49,52].

Contigs identified as Sapovirus GII (1/14) and GV (2/14) could not be typed using the Noronet Typing tool even though the complete genome was nearly assembled. GV is commonly linked to food-borne outbreaks [53,54,55], and some studies have shown a lack of detection of these types by conventional RT-PCR, while they can be detected when applying NGS [54,55]. Alignments of the complete genomes retrieved from the sequenced pools against the complete genomes and the VP1 region from reference strains showed the clustering of GII contigs with SaV GII.1b, whereas sequenced GV contigs clustered with GV.1 (Supplementary material 3).

### 3.3. Astroviridae

Human astroviruses (HAstV) are important agents causing acute gastroenteritis in children and have also been involved in outbreaks affecting adults [56]. Recently, astroviruses have also been associated with a wider array of diseases, including central nervous system alterations such as meningitis or acute flaccid paralysis [57,58]. The classification is currently based on the amino acid sequence of the capsid region (ORF2), dividing the HAsV in the “classical” genotypes (Mamastrovirus 1 (MAstV-1) species) and the “novel” recombinant ones (MAstV-6, 8, and 9 species) [22,59]. A summary of the astrovirus species detected is presented in Table 3.

Nearly the full genomes (6469–6652 bp) of four different astroviruses were retrieved. Human astrovirus species were found in all analyzed pools (Figure 1). HAstV-1 was detected in two pools and is the most common type associated with infantile gastroenteritis [60]. A HAstV-2 presenting a low homology over the full genome (85.7% identity) was detected in the S1-3A pool. During the development of the study, a commercially available immunochromatographic test was used to detect human astroviruses. In pooled samples “F”, this test was only used under specific circumstances (clinical signs supported an astrovirus infection, while samples “S” were all tested with this immunochromatographic test). Interestingly, among samples tested by the antigenic test, one result was inconclusive, which could be partially explained by the low homology of the detected astrovirus in this pool of samples. Phylogenetic trees with HAstV ORF2 complete sequences (Supplementary material 4a) and complete HAstV genomes (Supplementary material 4b) were constructed.

Nearly the full genome (6569 bp) of an astrovirus MLB-3 was detected in the F < 1 pool. This novel astrovirus was first described in 2014 in stools from children with acute diarrhea [61]. It has also been reported in asymptomatic patients [62] or involved in coinfections with other microorganisms that cause diarrhea, raising doubts regarding its role as a gastroenteritis causative agent [62,63].

### 3.4. Adenoviridae

Human adenovirus is a well-known pathogen in children as well as adults, producing a wide array of diseases, from gastroenteritis to conjunctivitis, respiratory infections, cystitis and meningitis. There are currently 104 recognized human adenovirus types according to the Human adenovirus working group (http://hadvwg.gmu.edu/) divided into seven different adenovirus species (A–G) with species D being the most abundant [64]. Sequencing has allowed the identification of several recombinant types, rapidly increasing the number of reported human adenoviruses in the literature [65,66].

Human adenovirus contigs were reported in pools F1-3A, S1-3B and F3-10. No human adenovirus species F (HAdV40 and 41), which are important etiological gastroenteritis agents in children [67], were detected in any of the pooled samples. This suggests a high specificity of immunochromatographic tests for HAdV species F. However, the full genome of a HAdV31 has been retrieved from the pool F3-10. The phylogenetic analysis of the sequenced human adenovirus clusters with HAdV31 reference type (Supplementary material 5). HAdV31 has been identified as a causative agent of acute gastroenteritis in children despite not belonging to HAdV species F [68].

Other HAdV type partial sequences have been identified based on the hexon, penton and fiber analysis. These adenoviruses might belong to species A (HAdV18, HAdV61, HAdV61, HAdV31), B (HAdV3, HAdV66), and C (HAdV89). Human adenoviruses are known to produce permanent infections in symptomatic and asymptomatic individuals. Hence, the detection of fragments of the adenovirus genomes could be indicative of the presence of persistent HAdV infections [69,70]. However, the lower number of contigs associated to other than HAdV31 and HAdV F species make them unlikely to be the causative agents of acute gastroenteritis.

### 3.5. Reoviridae

Rotavirus (RV) reads were detected in pools F < 1, S < 1A, S < 1B and S3-10. All detected RV belonged to the genotype G2P[4]. This genotype is one of the six most commonly genotypes detected worldwide, together with G1P[8], G3P[8], G4P[8], G9P[8], and G12P[8] [71,72]. RV has been identified as the main leading fatal etiologic agent for diarrhea in children less than five years old, causing 128,515 deaths in 2016 [1].

In the pool of fecal samples F < 1, S < 1A, and S < 1B, reads from 9 out of the 11 RVA genes were as follows: six structural protein (VP) coding genes and three nonstructural (NSP) coding genes. In the genotyping analysis, R2, C2, M2, P[4], I2, and G2, referred to as VP1, VP2, VP3, VP4, VP6, and VP7, respectively, were detected, and N2, T2, E2, referred to as NSP2, NSP3, and NSP4, respectively, were detected for the NSP genotypes [73]. In pool S3-10, reads of the genes coding for VP2, VP3, VP4, VP6, and VP7 with the same genotype pattern observed previously were found: C2, M2, P(4), I2, and G2. RVA detected in the present study showed high similarity (99.5–100%) to the genotype G2P(4) RVA strains circulating in Europe, Asia, and South America in the last decade for both pooled samples (data not shown). In this study, no RVA sequences revealed a close nucleotide similarity with the two commercially anti-RVA vaccines (Rotarix^®^ and Rotateq^®^) available worldwide. Together, these results suggest that children probably acquired RVA strains circulating in Barcelona from 2012 to 2014, having no correlation with an anti-RVA vaccination. A study conducted between 2015 and 2016 revealed the presence of RVA in high viral loads in sewage samples from Barcelona. The authors also found no evidence of RVA vaccine-related strains in the sewage samples [74]. These results suggest that RVA is widespread in the community, since the population excretes RVA and the children still require healthcare assistance due to gastroenteritis associated with RVA.

Immunochromatographic tests for RVA diagnosis have been a useful tool to provide quick and cost-effective tests for routine clinical analyses at the expense of low sensitivity when compared to molecular methods such as RT-PCR [75,76]. The low number of reads obtained within the pools might be explained by three important facts: (a) the samples could have low levels of virus, since all were negative in the commonly used test; (b) individual patient samples were pooled, diluting the number of viral reads present in positive patients; (c) dsRNA viruses have been shown to be difficult to detect using current NGS techniques [77].

### 3.6. Other Viral Species Identified in Fecal Samples

Detected picornaviruses have been taxonomically classified according to their genera: *Enterovirus, Kobuvirus, Parechovirus and Cardiovirus*. Enterovirus and non-enterovirus picornaviruses have been summarized in Supplementary materials 6–8.

Enterovirus (EV) causes a wide range of diseases in children, such as hand-foot-mouth disease (HFMD), viral meningitis and encephalitis, acute flaccid paralysis, myocarditis, common cold or neonatal sepsis. It is also reported to be present in asymptomatic infections or coinfections with another microorganism both in children and adults [78].

The EV genus is subdivided by its capsid sequence (VP1) in 13 species, with EV-A, EV-B, EV-C, EV-D, rhinovirus A (RV-A), RV-B and RV-C infecting humans [79,80]. Enterovirus from species EV-A and EV-B with RV-A and RV-B were detected by NGS in the pooled samples. An exhaustive list containing the typed enterovirus and rhinovirus using the RIVM enterovirus typing tool is provided in Supplementary material 6.

Rhinoviruses A (HRV-A) and B (HRV-B) were present in all the pools analyzed. These species are the most important etiological agents of common cold, with a high incidence of infections in early childhood, mainly affecting the upper respiratory airways, with HRV-A being the most prevalent [81]. In the S2 pool, nearly the full genome of HRV-A78 was obtained (6499 bp). HRV is one of the predominant circulating species of HRV-A in children [82] and has been previously associated with pneumonia [83].

Members of the genus *Parechovirus* (HPeV) are common causes of aseptic meningitis in children and mild gastrointestinal and respiratory diseases [84]. Currently, more than 18 different types have been recognized to infect humans according to the HPeV ICTV taxonomy group, all of which cluster under HPEV-A species (https://talk.ictvonline.org/ictv-reports/ictv_online_report/positive-sense-rna-viruses/picornavirales/w/picornaviridae/693/genus-parechovirus). Sequences from HPeV A were detected in pools associated with younger patients (<3 years). Phylogenetic analysis allowed their classification as HPeV-1 and HPeV-6 (Supplementary material 8).

The *Cardiovirus* genus was first associated with rodent infections, until their role in human pathogenicity was described in 2007 [85]. There are currently three recognized species, *Cardiovirus* A, *Cardiovirus* B and *Cardiovirus* C. More than 11 genotypes of Cardio-virus B species are associated with human infection, with Saffold viruses (SAFV) 2 and 3 being the most prevalent.

Almost the whole genome of a *Cardiovirus* B member, genotyped as SAFV-2, was sequenced in pool F > 10 from affected individuals above 10 years old (Supplementary material 7). SAFV are not routinely tested in clinical samples, but recent studies have reported a common presence in gastroenteritis patients [86,87] and in raw sewage [88,89].

The genus *Kobuvirus* is composed of six recognized *Aichi virus* (AiV) viral species (AiVA-AiVF) that are classified according to their amino acid homology in the polyprotein, P1 2C and 3CD regions (https://talk.ictvonline.org/ictv-reports/ictv_online_report/positive-sense-rna-viruses/picornavirales/w/picornaviridae/686/genus-kobuvirus). Members of the AiV A and B species have been recognized as human pathogens causing gastroenteritis outbreaks [90,91,92]. Members within this genus are considered zoonotic and have the potential to infect dogs, cats or goats [93].

Two different Aichi virus contigs were detected in pool S3-10. The alignments of the retrieved contigs were phylogenetically related to AiV A which has been associated with acute gastroenteritis.

Picobirnavirus-related contigs were detected in most of the pools analyzed except F < 1 and S3-10. Previously, picobirnavirus has been considered a putative pathogenic viral species present in the respiratory and digestive tract of humans and other animals, including pigs, cows, dogs and otters. However, recent research has suggested that picobirnavirus might be a prokaryotic virus because the ribosomal binding site (RBS) motif is conserved within its genome. This motif has been detected in all available picobirnavirus sequences and is commonly enriched in all viral families that infect prokaryotes, but not in eukaryotic viral families [94].

Contigs belonging to primate bocaparvovirus 2, formerly known as Human Bocavirus 2, were found in pool S6. These viral species have been previously found in stool samples from gastroenteritis-related studies in young children with variable prevalence depending on the area of study and year of sample collection [95].

A diverse number of ssDNA circular viruses belonging to the *Anelloviridae* and *Circoviridae* families have been detected among the different pools analyzed. The family *Anelloviridae* includes the Torque teno virus (TTV), Torque teno midi virus (TTMDV) and mini virus Torque teno virus (TTMV). The detection of anellovirus species in clinical samples does not seem unlikely, as it is estimated that 70–90% of the human population carries the virus in loads that range from 10^3^ to 10^6^ genomic copies/mL plasma [96]. None of the different torque teno virus species known to date have been directly associated with disease [97].

However, the leading role in acute gastroenteritis of the discussed viral agents under this subsection is at least controversial, as most of them can replicate actively in the intestinal tract [98], have been co-detected with other well stablished gastrointestinal viral pathogens such as RV, NoV, SaV or HAdV [78,86,92,99] or are commonly detected in non-symptomatic control samples [97,100].

Further studies focusing on these specific families in non-pooled samples are needed to clarify their involvement in acute gastroenteritis either as monoinfection or coinfection pathogens.

### 3.7. Target Enrichment Sequencing

A target enrichment approach (TES) was performed in one pool (F1-3A) to evaluate its usefulness for the detection of gastroenteritis viral agents compared with untargeted metagenomics (UVM) applied in this study. The application of a target enrichment probe-based capture method, prior to sequencing, has been demonstrated to overcome the low sensitivity or the lack of sequencing depth in complex samples with large viral diversity [12,101].

**Figure 3:**
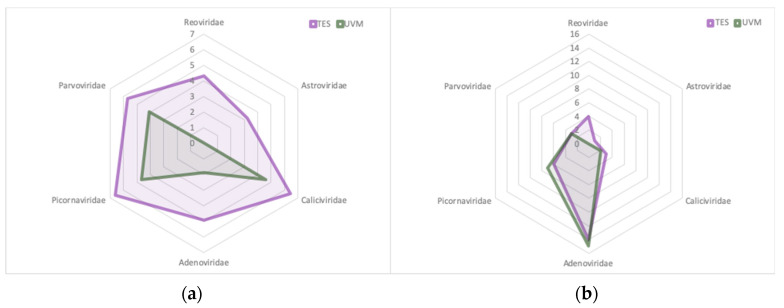
Comparison of the number of viral reads (**a**) and contigs (**b**) from families with members related to gastroenteritis with Target Enrichment Sequencing (TES) and Untargeted Viral Metagenomics (UVM) in the F1-3A pool. The number of reads is represented as log10.

Comparing both methodologies and focusing on viral families of interest regarding gastroenteritis etiology, TES enabled the detection of a larger number of viral reads (Figure 3a), allowing the detection of *Reoviridae* members, which were not detected with the UVM approach. Additionally, TES allowed the typing of the hexon, penton and fiber genes of a HAdV-31 that could not be typed by UVM. In contrast, when analyzing these reads as contigs, the differences were reduced to obtain the same number of contigs by applying both methodologies to the detected families (Figure 3b). This observation could be related to the sequence fragmentation and diversity of the sample, the absence of sequences from different regions of each viral assignation, or the absence of regions to which the probes are hybridizing. This phenomenon would result in a large number of reads from the same genome region and a reduction of diversity, but an increase in accuracy of the contig sequence.

The application of TES allowed the detection of 4 RV contigs in the sample pool analyzed that were not previously detected in the UVM pool. The detected rotavirus sequences belonged to genotype G2P[4], the predominant RV circulating in Europe between 2010 and 2019 [71,72,102]. As observed for other detected RV sequences in the study, none of the contigs was related to the two commercially available rotavirus vaccines.

## 4. General Discussion and Conclusions

Viral metagenomics, in combination with or without a viral enrichment approach, has the potential to uncover any viral species that might be present in a given sample. These methodologies are not primer-dependent methods; hence, they detect viral species that might be undetected by using conventional molecular methodologies such as RT-PCR or RT-qPCR. The use of primer-independent methodologies represents an important advantage in detecting new viral pathogens (i.e., SARS-CoV-2) or recombinant/reassortant types. These methodologies are especially relevant for viral species, as viruses are prone to have high mutation rates and to recombine/reassort their genomes as an evolutionary mechanism. In addition, the gastrointestinal tract is a complex ecosystem, and the evolution and severity of acute gastroenteritis might be conditioned by the presence of coinfections that could be masked by the application of single pathogen detection methods such as immunochromatography or real-time PCR. However, a large number of sequences obtained by metaviromics cannot be taxonomically classified by using reference databases and are considered viral dark matter, requiring a more exhaustive analysis [103,104]. The use of a viral-enriched approach (TES) increased the number of viral reads up to 74%, in contrast to the 23% viral reads detected without enrichment in the same pooled sample. However, despite the enrichment applied, a wider diversity of viral species could be detected without the viral enrichment with the exception of rotavirus A, which was not detected without applying TES.

According to the number of reads detected, the most abundant sequences from vertebrate viral families were from *Astroviridae*-related members, which were identified in all age groups, suggesting the need to include systematic molecular testing for this particular viral family independently of the patient age. Following AstV, NoV and SaV present an important number of reads. At the time of sample collection, clinical screening did not include any molecular assay to detect sapoviruses; thus, their detection using NGS seems reasonable. Sapoviruses have been identified as an increasing etiological agent for human gastroenteritis, and their screening in the clinical setting should be systematically performed at least for children under 10 years of age. Human noroviruses were detected in all pools analyzed despite most samples having been previously tested by conventional RT-PCR in clinical settings. Some of these NoV might have escaped detection by RT-PCR due to the presence of mismatches in primer annealing zones. Additional in-silico analysis of the NGS-retrieved sequence showed mismatches in the primer binding region, highlighting the utility of primer-independent methodologies to target viruses with a high mutation rate. Interestingly, NoV GIV, a minority NoV species related to gastroenteritis, was detected by NGS and confirmed in three patients by RT-qPCR. The inability to detect this specific NoV genogroup is expected because it is not routinely tested. Samples for which the NoVs test was ruled out according to clinical evaluation showed the presence of both NoV GI and GII. Therefore, we recommend the inclusion of NoVGI, GII and GIV in the systematic testing of all acute gastroenteritis cases.

HAdV not belonging to species F were detected in patients less than 10 years old. The use of immunochromatographic tests targeting adenovirus species F (HAdV 40 and 41) might present a lower sensitivity towards other adenoviruses (i.e., HAdV species A or C), which could be the etiological agents for some acute gastroenteritis such as the detected HAdV31. Nevertheless, HAdV are known to produce a wide array of infections including asymptomatic persistent infections of the gastrointestinal tract, raising doubts about their involvement in acute gastroenteritis. Similarly, human enteroviruses and other related picornaviruses (such as parechovirus and cardiovirus) can produce a wide array of clinical outcomes but are also persistently excreted by healthy asymptomatic individuals. Thus, without any additional clinical information, their detection in clinical samples might result from a coinfection with another gastroenteritis virus or simply as a subclinical infection. In this sense, the application of viral metagenomics has emerged as a promising tool contributing to increased knowledge of excreted viruses and their role in human pathogenesis.

Finally, a small proportion of reads could be taxonomically assigned to the family *Reoviridae*, which includes HRV. According to the phylogenetic analyses, the identified RV were not related with any of the existent vaccine strains; hence, their presence in the analyzed pools suggests their implication in the clinical gastroenteritis despite the low genome coverage and small number of reads retrieved from this particular viral family in this study. Similarly to HAdV, the small number of obtained reads could be explained by an inefficient tagging of double-stranded genomes during the retro-transcription or sequenase steps resulting in low coverage and the small number of reads assigned to this viral family. Specific protocols to increase the sensitivity towards double-stranded genomes (dsDNA and dsRNA) might need to be applied to explore the viral diversity within these genomic conformations [77].

## Figures and Tables

**Table 1:** Summary of the samples composing each sequenced pool according to age criteria and the performed conventional diagnostic assays in the clinical center. The results of the bacterial and viral parameters are expressed as the “number positive samples/number samples tested”. For human adenovirus, human rotavirus and human astrovirus, a commercially available immunochromatographic test was employed (ONE STEP Adenovirus CARD TEST, ONE STEP Rotavirus CARD TEST and ONE STEP Astrovirus CARD TEST, Certest Biotec S.L). For norovirus GI and GII, conventional RT-PCR was used [9].

			Bacterial Parameters							Viral Parameters				
Pool	Age	N	*Salmonella* sp.	*Campylobacter* sp.	*Shigella* sp.	*Yersinia* sp.	*Aeromonas* sp.	*Verotoxigenic E. coli (VTEC)*	*Vibrio* sp.	Rotavirus	Adenovirus	Astrovirus	Norovirus GGI	Norovirus GGII
F < 1	<1	10	0/9	0/9	0/9	0/9	0/0	0/0	0/0	0/10	0/10	0/10	0/10	0/10
S < 1A		9	0/9	0/9	0/9	0/9	0/7	0/0	0/8	0/9	0/9	0/9	0/0	0/0
S < 1B		9	0/9	0/9	0/9	0/9	0/8	0/0	0/8	0/9	0/9	0/9	0/1	0/1
F1-3A	1–3	23	0/23	0/23	0/23	0/23	0/0	1/1	0/1	0/23	0/23	0/23	0/23	0/23
S1-3A		14	0/13	0/13	0/13	0/13	0/11	0/1	0/11	0/13	0/13	0/13	0/1	0/1
S1-3B		14	0/14	0/14	0/14	0/14	0/7	0/0	0/8	0/14	0/14	0/14	0/1	0/1
S1-3C		13	0/14	0/14	0/14	0/14	0/8	0/0	0/11	0/14	0/14	0/14	0/0	0/0
F3-10	3–10	13	0/13	0/13	0/13	0/13	0/0	0/0	0/0	0/12	0/12	0/13	0/13	0/13
S3-10		10	0/9	0/9	0/9	0/9	0/9	0/0	0/9	0/1	0/10	0/10	0/0	0/0
F > 10	>10	9	0/9	0/9	0/9	0/9	0/0	0/1	0/1	0/8	0/8	0/9	0/9	0/9

**Table 2:** Characterization of *Caliciviridae* contigs detected in the gastroenteritis pools with the norovirus typing tool by RIVM (v 2.0) using the dual system assessing the polymerase (ORF1) and the capsid region (ORF2). NT: non-typable by the RIVM Norovirus Genotyping Tool.

Pool	Contig Length	Classification	Polymerase Genotype	Capsid Genotype	BLAST Score
F < 1	7521	Sapovirus GV	NT	GV.1	98.03
S < 1A	7440	Norovirus GII	GII.P21 (GII.Pb)	GII.3	80.95
F1-3A	7187	Norovirus GIV	NT	NT	77.26
	4827	Sapovirus GI	NT	GI.2	73.61
S1-3A	5978	Norovirus GII	GII.P31 (GII.Pe)	GII.2	85.10
	1107	Norovirus GII	NT	NT	88.89
	7292	Sapovirus GII	NT	GII.1b	74.37
	550	Sapovirus GI	NT	NT	74.27
	376	Sapovirus GI	NT	NT	74.86
	698	Sapovirus GI	NT	NT	76.72
	2109	Sapovirus GI	NT	NT	72.10
	859	Sapovirus GI	NT	GI.3	78.06
	1321	Sapovirus GI	NT	GI.3	71.14
S1-3B	6808	Norovirus GII	GII.P17	GII.17	75.56
F3-10	7384	Norovirus GII	GII.P4 New_Orleans_2009	GII.4 New_Orleans_2009	84.54
	7521	Sapovirus GV	NT	GV.1	98.08
S3-10	3533	Norovirus GI	NT	NT	
	2065	Norovirus GI	NT	GI.2	69.38
	798	Norovirus GI	GI.P2	GI.2	77.26
	1551	Sapovirus GI	NT	NT	75.02
	1059	Sapovirus GI	NT	NT	72.61
	345	Sapovirus GI	NT	GI.3	66.47
	349	Sapovirus GI	NT	GI.3	73.99
F > 10	924	Norovirus GIV	NT	NT	98.70
	576	Norovirus GIV	NT	NT	98.44
	1249	Norovirus GIV	NT	NT	99.25
	375	Norovirus GIV	NT	NT	98.67

**Table 3:** Characterization of human astrovirus contigs detected in the tested fecal pools.

Pool	Contig Length	Classification	ORF2 Genotype	% ORF2 Identity	% ORF2 Coverage	% Genome Identity	% Genome Coverage
F < 1	6569	Mamastrovirus 6	MLB-3	97.8	100	98.1	99.8
S1-3A	6642	Mamastrovirus 1	HAstV-2	92.7	100	85.7	97.6
F3-10	6652	Mamastrovirus 1	HAstV-1	95.4	100	94.1	97.6
F > 10	6651	Mamastrovirus 1	HAstV-1	95.1	100	94.0	97.6

## Data Availability

The datasets generated during the current study are available in zenodo under the DOI number https://doi.org/10.5281/zenodo.4161275 (https://zenodo.org/record/4161275#.X6KAjyySlPY).

## Supplementary Materials

Supplementary Materials can be found at https://www.mdpi.com/1999-4915/12/12/1432/s1.
