# Supplementary material for: Unveiling Viruses Associated with Gastroenteritis Using a Metagenomics Approach
Source: Viruses. 2020 Dec 13;12(12):1432. doi: 10.3390/v12121432 (PMC7764520; doi:10.3390/v12121432)
Supplement: Supplementary file 1 [file viruses-12-01432-s001.zip › Supplementary files.pdf]

Supplementary material 1. Metagenomic sequencing summary statistics obtained with genome detective.

Sample	Initial paired end reads	Removed reads after quality (percentage)	Used reads	Non-viral reads (percentage)	reads used for assembly	Mapped reads into contigs
F<1	6,095,990	468,766 (7%)	5,627,224	2,867,732 (50%)	2,759,492	2,364,300
F1-3A	5,613,532	220,524 (3%)	5,393,008	4,161,306 (77%)	1,231,702	487,708
F3-10	9,237,546	513,822 (5%)	8,723,724	1,049,524 (12%)	7,674,200	7,463,778
F>10	12,265,116	655,736 (5%)	11,609,380	4036,566 (34%)	7,572,814	7,414,041
S<1A	1,982,130	119,564 (6%)	1,862,566	1,049,222 (56%)	813,344	873,829
S<1B	1,258,278	69,804(5%)	1,188,474	850,264 (71%)	338,210	199,647
S1-3A	1,549,150	60,662 (3%)	1,488,488	819,480 (55%)	669,008	514,749
S1-3B	1,037,992	41,840 (4%)	996,152	725,326 (72%)	270,826	85,046
S1-3C	881,740	37,730 (4%)	844,010	379,784 (44%)	464,226	17,425
S3-10	2,419,026	70,822 (2%)	2,348,204	1,387,786 (59%)	960,418	578,919

Supplementary material 3. Phylogenetic trees of sapovirus contigs. Trees were constructed by using tree view software within Geneious (software version 11.0 (https://www.geneious.com) maximum-likelihood (Tamura-Nei model, Bootstrap of 1,000 replicates) analysis. Phylogenetic trees with the full sapovirus types (a), full length VP1 (b), complete genomes are presented. The labels on the branch indicate the number of substitutions per site.

a.

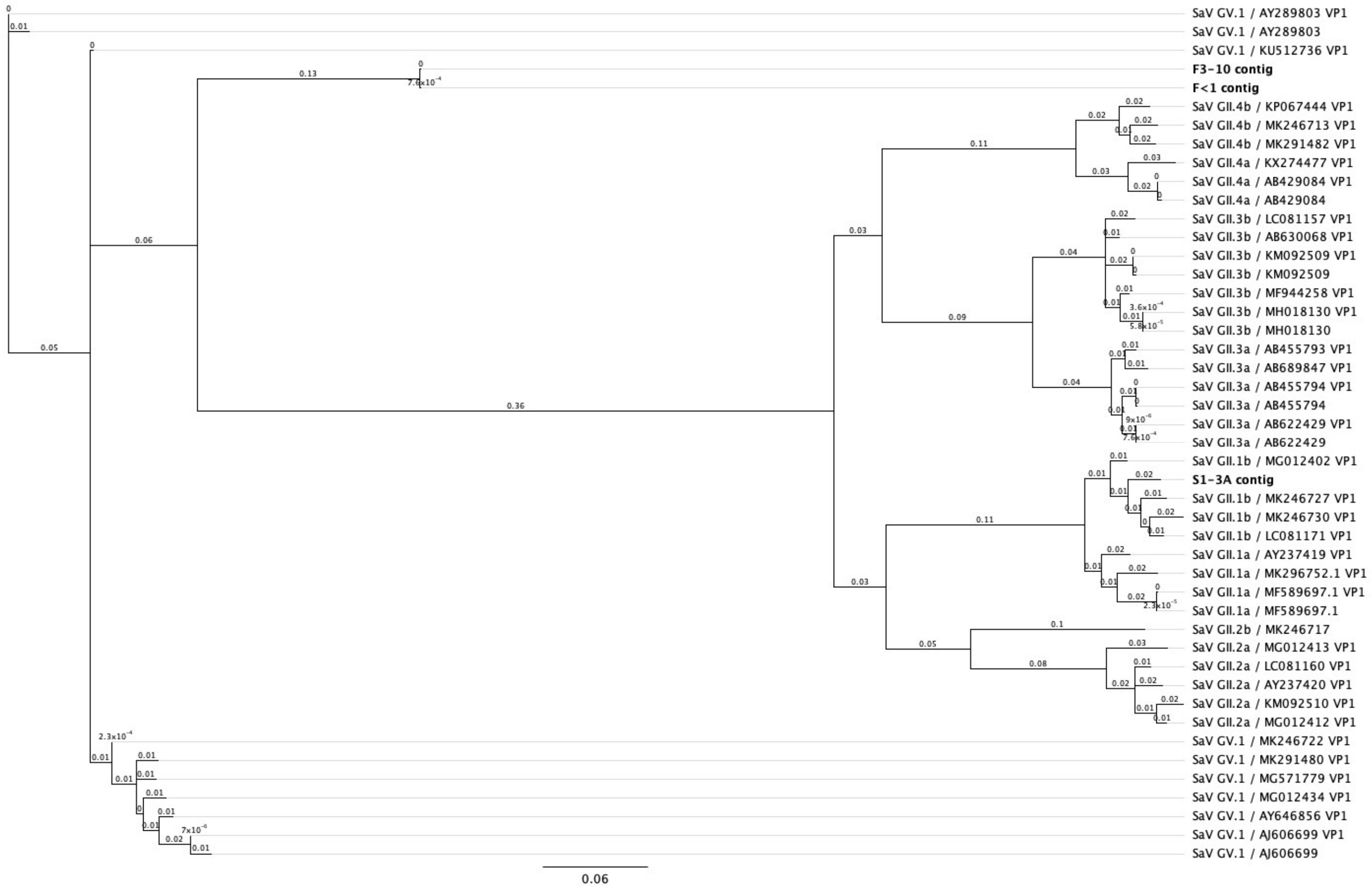

b.

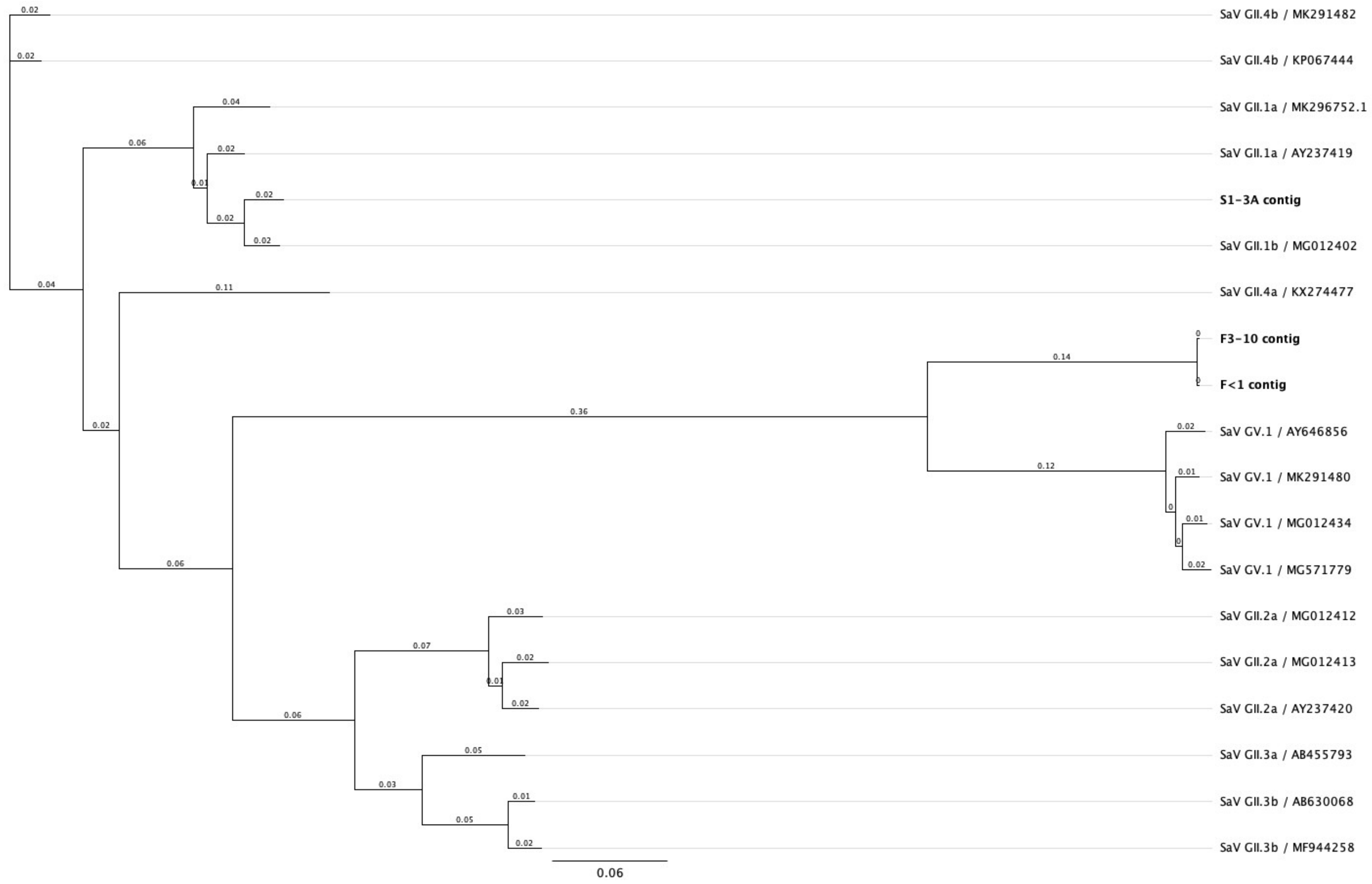

SaV GII.4b / MK291482
SaV GII.4b / KP067444
SaV GII.1a / MK296752.1
SaV GII.1a / AY237419
S1-3A contig
SaV GII.1b / MG012402
SaV GII.4a / KX274477
F3-10 contig
F<1 contig
SaV GV.1 / AY646856
SaV GV.1 / MK291480
SaV GV.1 / MG012434
SaV GV.1 / MG571779
SaV GII.2a / MG012412
SaV GII.2a / MG012413
SaV GII.2a / AY237420
SaV GII.3a / AB455793
SaV GII.3b / AB630068
SaV GII.3b / MF944258
0.06

Supplementary material 4. Phylogenetic trees of mamastrovirus contigs. Trees were constructed by using tree view software within Geneious (software version 11.0 (https://www.geneious.com) maximum-likelihood (Tamura-Nei model, Bootstrap of 1,000 replicates) analysis. Phylogenetic trees with the full mamastrovirus types (a), full length ORF2 (b), complete genomes are presented. The labels on the branch indicate the number of substitutions per site.

a.

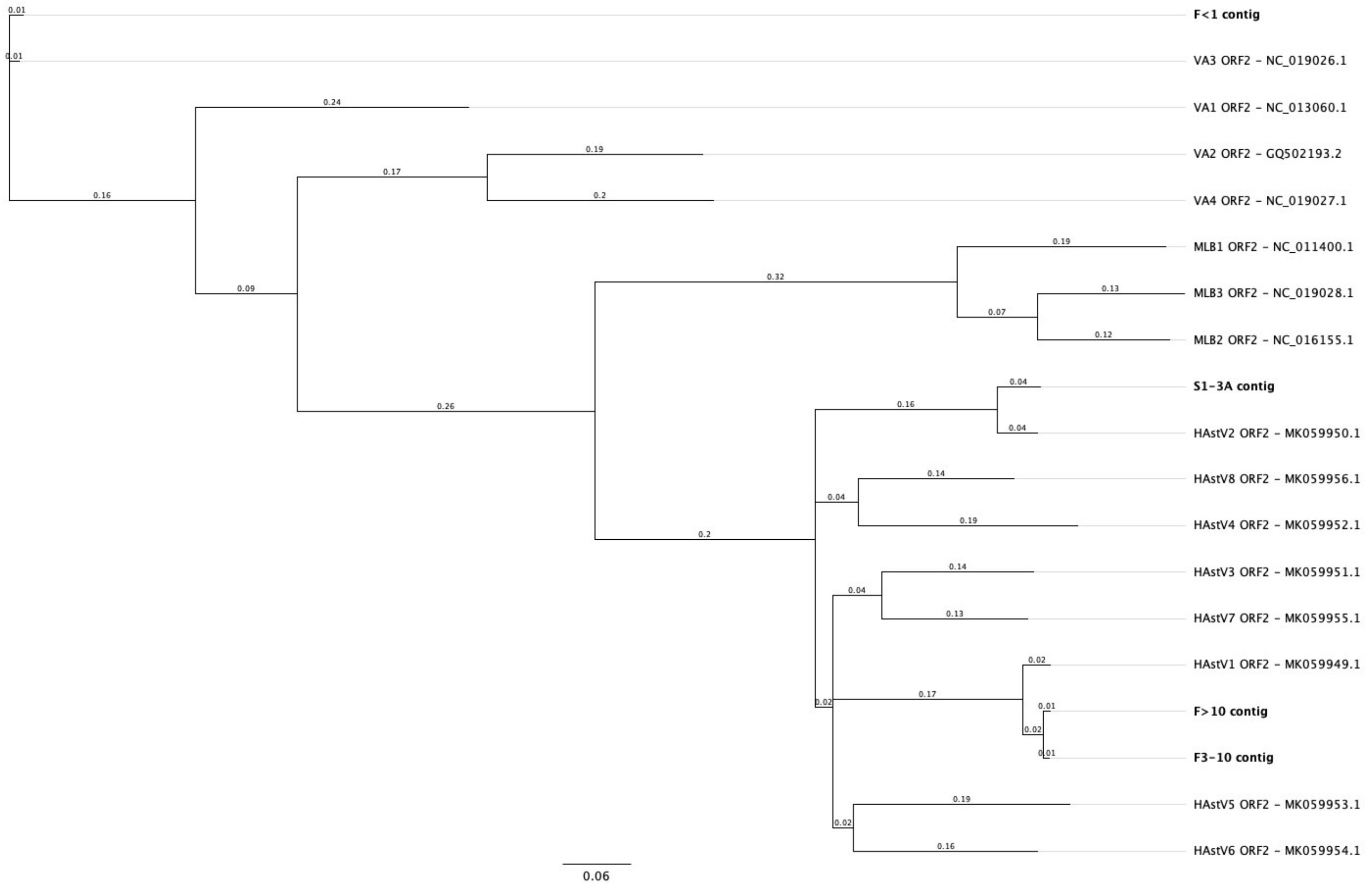

b.

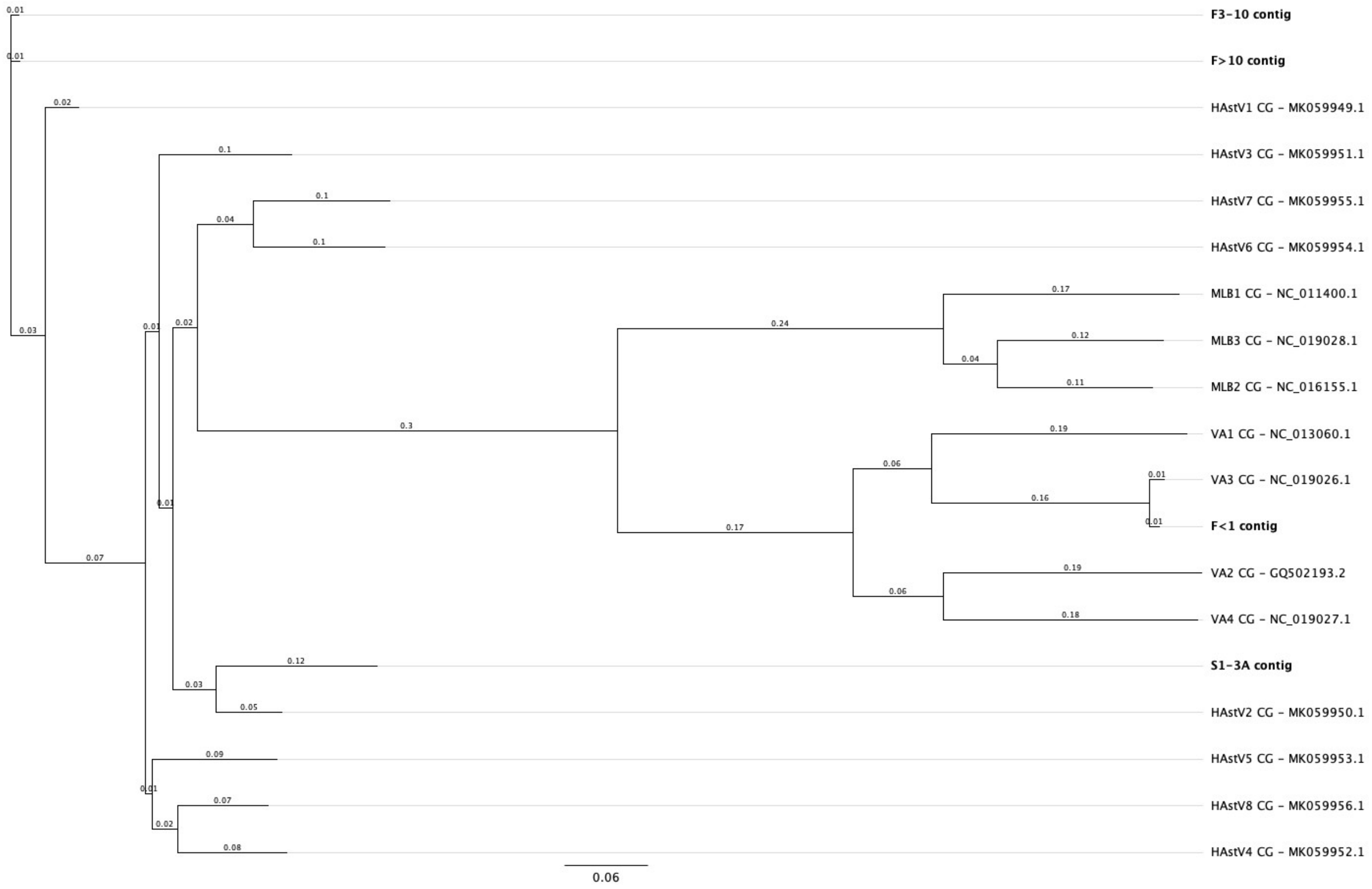
F3-10 contig
F>10 contig
HAstV1 CG - MK059949.1
HAstV3 CG - MK059951.1
HAstV7 CG - MK059955.1
HAstV6 CG - MK059954.1
MLB1 CG - NC_011400.1
MLB3 CG - NC_019028.1
MLB2 CG - NC_016155.1
VA1 CG - NC_013060.1
VA3 CG - NC_019026.1
F<1 contig
VA2 CG - GQ502193.2
VA4 CG - NC_019027.1
S1-3A contig
HAstV2 CG - MK059950.1
HAstV5 CG - MK059953.1
HAstV8 CG - MK059956.1
HAstV4 CG - MK059952.1
0.06

Supplementary material 5

Accession numbers of reference adenovirus types were retrieved from the Human Adenovirus Working Group (http://hadvwg.gmu.edu/). Full adenovirus genomes or the annotated regions corresponding to the hexon, penton and fiber were extracted and aligned with MUSCLE. Phylogenetic tree were constructed by using tree view software within Geneious (software version 11.0 (https://www.geneious.com) maximum-likelihood (Tamura-Nei model, Bootstrap of 1,000 replicates) analysis. Phylogenetic trees with the full adenovirus reference types (a), using the hexon (b), penton (c) and fiber (d) are presented. The labels on the branch indicate the number of substitutions per site.

a)

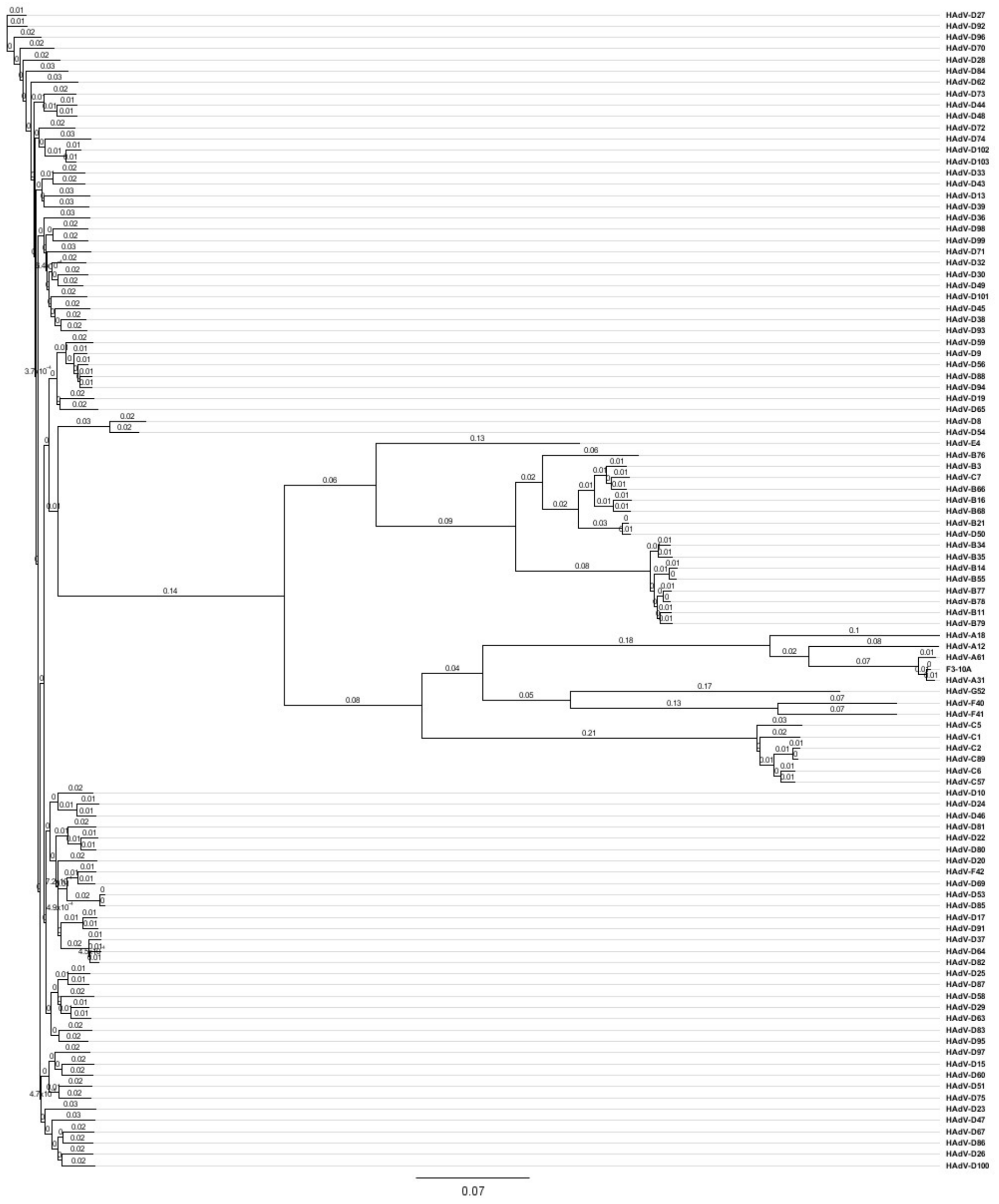

b)

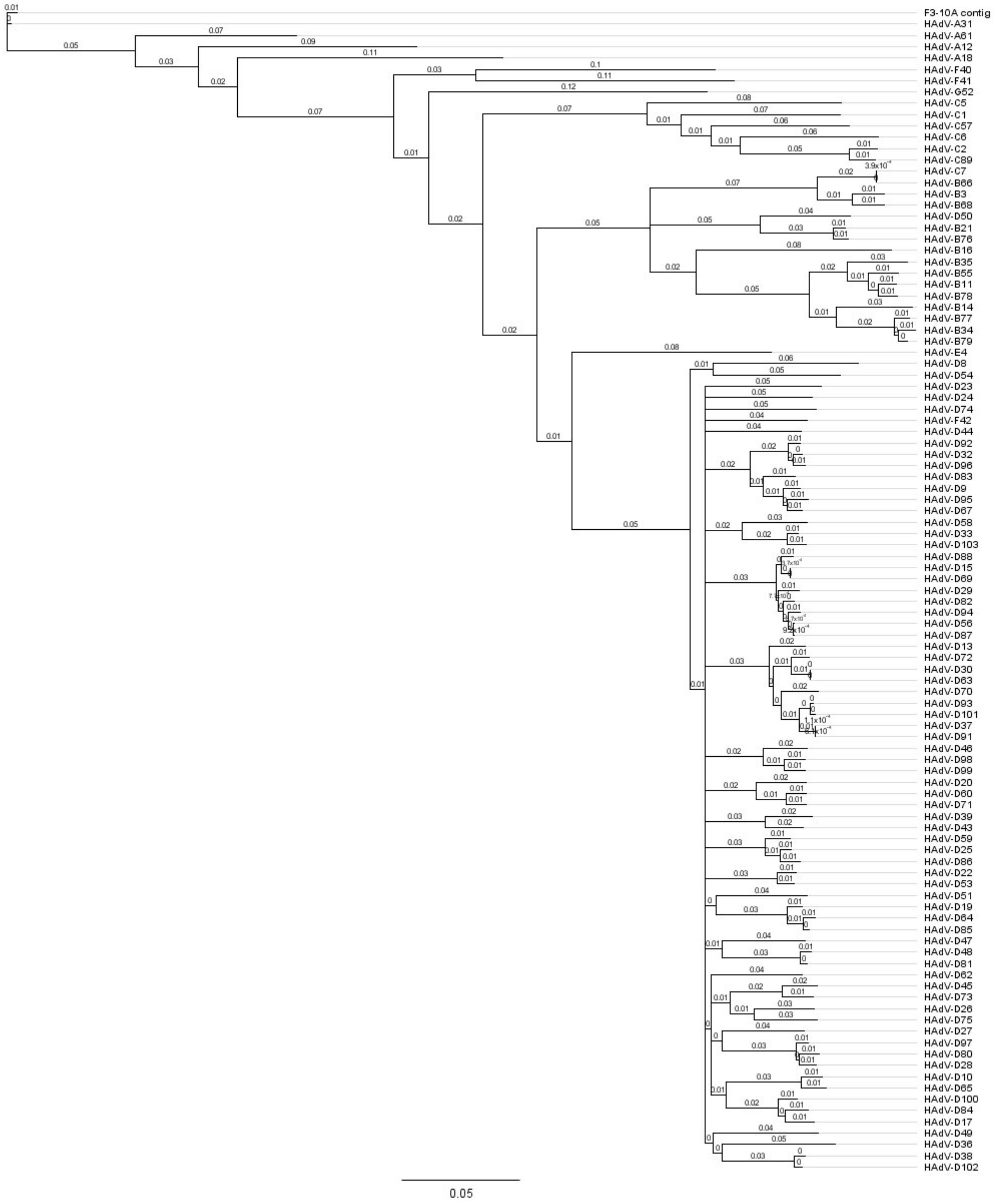
F3-10A contig
HAdV-A31
HAdV-A61
HAdV-A12
HAdV-A18
HAdV-F40
HAdV-F41
HAdV-G52
HAdV-C5
HAdV-C1
HAdV-C57
HAdV-C6
HAdV-C2
HAdV-C89
HAdV-C7
HAdV-B66
HAdV-B3
HAdV-B68
HAdV-D50
HAdV-B21
HAdV-B76
HAdV-B16
HAdV-B35
HAdV-B55
HAdV-B11
HAdV-B78
HAdV-B14
HAdV-B77
HAdV-B34
HAdV-B79
HAdV-E4
HAdV-D8
HAdV-D54
HAdV-D23
HAdV-D24
HAdV-D74
HAdV-F42
HAdV-D44
HAdV-D92
HAdV-D32
HAdV-D96
HAdV-D83
HAdV-D9
HAdV-D95
HAdV-D67
HAdV-D58
HAdV-D33
HAdV-D103
HAdV-D88
HAdV-D15
HAdV-D69
HAdV-D29
HAdV-D82
HAdV-D94
HAdV-D56
HAdV-D87
HAdV-D13
HAdV-D72
HAdV-D30
HAdV-D63
HAdV-D70
HAdV-D93
HAdV-D101
HAdV-D37
HAdV-D91
HAdV-D46
HAdV-D98
HAdV-D99
HAdV-D20
HAdV-D60
HAdV-D71
HAdV-D39
HAdV-D43
HAdV-D59
HAdV-D25
HAdV-D86
HAdV-D22
HAdV-D53
HAdV-D51
HAdV-D19
HAdV-D64
HAdV-D85
HAdV-D47
HAdV-D48
HAdV-D81
HAdV-D62
HAdV-D45
HAdV-D73
HAdV-D26
HAdV-D75
HAdV-D27
HAdV-D97
HAdV-D80
HAdV-D28
HAdV-D10
HAdV-D65
HAdV-D100
HAdV-D84
HAdV-D17
HAdV-D49
HAdV-D36
HAdV-D38
HAdV-D102
0.05

c)

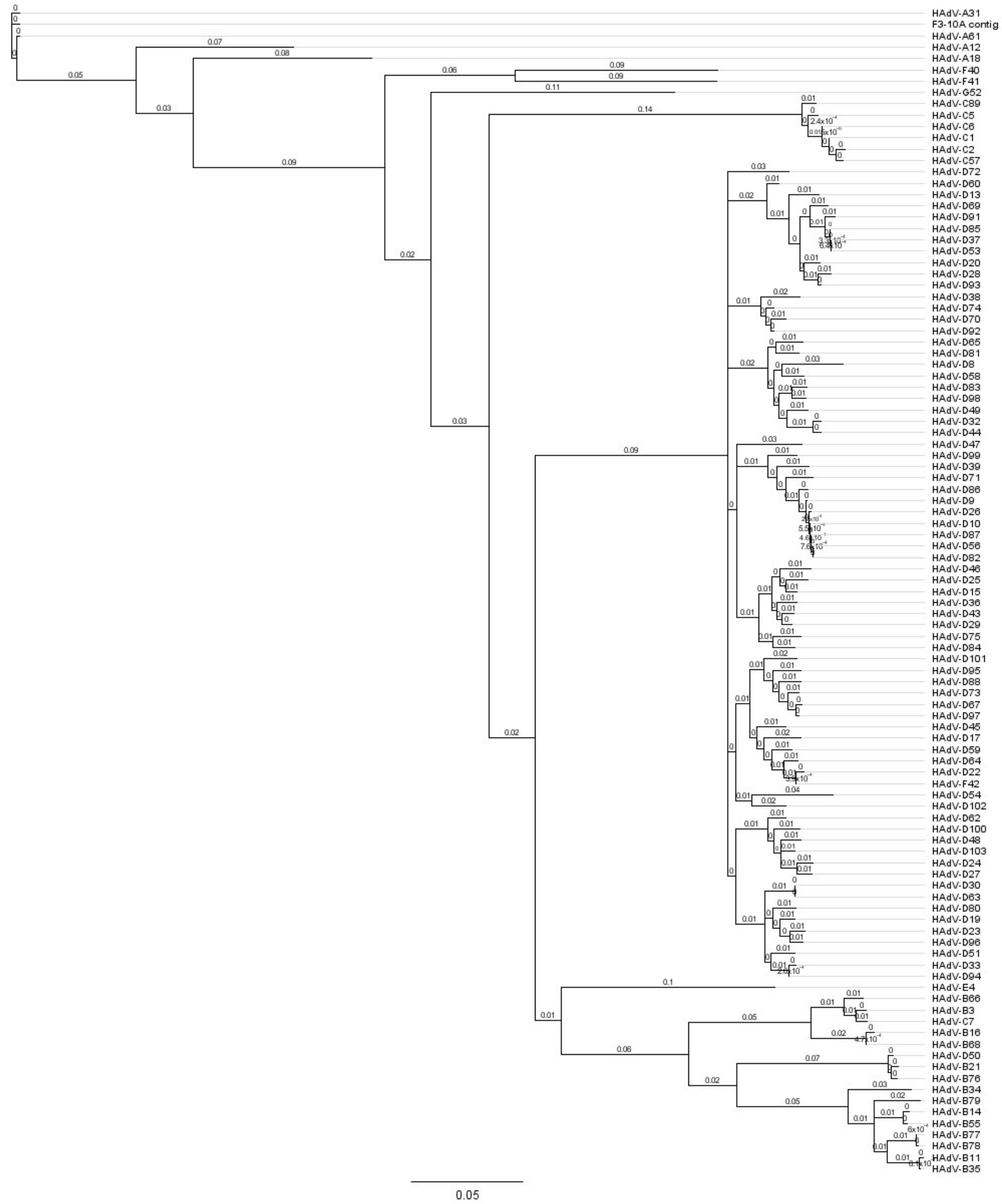
HAdV-A31
F3-10A contig
HAdV-A61
HAdV-A12
HAdV-A18
HAdV-F40
HAdV-F41
HAdV-G52
HAdV-C89
HAdV-C5
HAdV-C6
HAdV-C1
HAdV-C2
HAdV-C57
HAdV-D72
HAdV-D60
HAdV-D13
HAdV-D69
HAdV-D91
HAdV-D85
HAdV-D37
HAdV-D53
HAdV-D20
HAdV-D28
HAdV-D93
HAdV-D38
HAdV-D74
HAdV-D70
HAdV-D92
HAdV-D65
HAdV-D81
HAdV-D8
HAdV-D58
HAdV-D83
HAdV-D98
HAdV-D49
HAdV-D32
HAdV-D44
HAdV-D47
HAdV-D99
HAdV-D39
HAdV-D71
HAdV-D86
HAdV-D9
HAdV-D26
HAdV-D10
HAdV-D87
HAdV-D56
HAdV-D82
HAdV-D46
HAdV-D25
HAdV-D15
HAdV-D36
HAdV-D43
HAdV-D29
HAdV-D75
HAdV-D84
HAdV-D101
HAdV-D95
HAdV-D88
HAdV-D73
HAdV-D67
HAdV-D97
HAdV-D45
HAdV-D17
HAdV-D59
HAdV-D64
HAdV-D22
HAdV-F42
HAdV-D54
HAdV-D102
HAdV-D62
HAdV-D100
HAdV-D48
HAdV-D103
HAdV-D24
HAdV-D27
HAdV-D30
HAdV-D63
HAdV-D80
HAdV-D19
HAdV-D23
HAdV-D96
HAdV-D51
HAdV-D33
HAdV-D94
HAdV-E4
HAdV-B66
HAdV-B3
HAdV-C7
HAdV-B16
HAdV-B68
HAdV-D50
HAdV-B21
HAdV-B76
HAdV-B34
HAdV-B79
HAdV-B14
HAdV-B55
HAdV-B77
HAdV-B78
HAdV-B11
HAdV-B35
0.05

d)

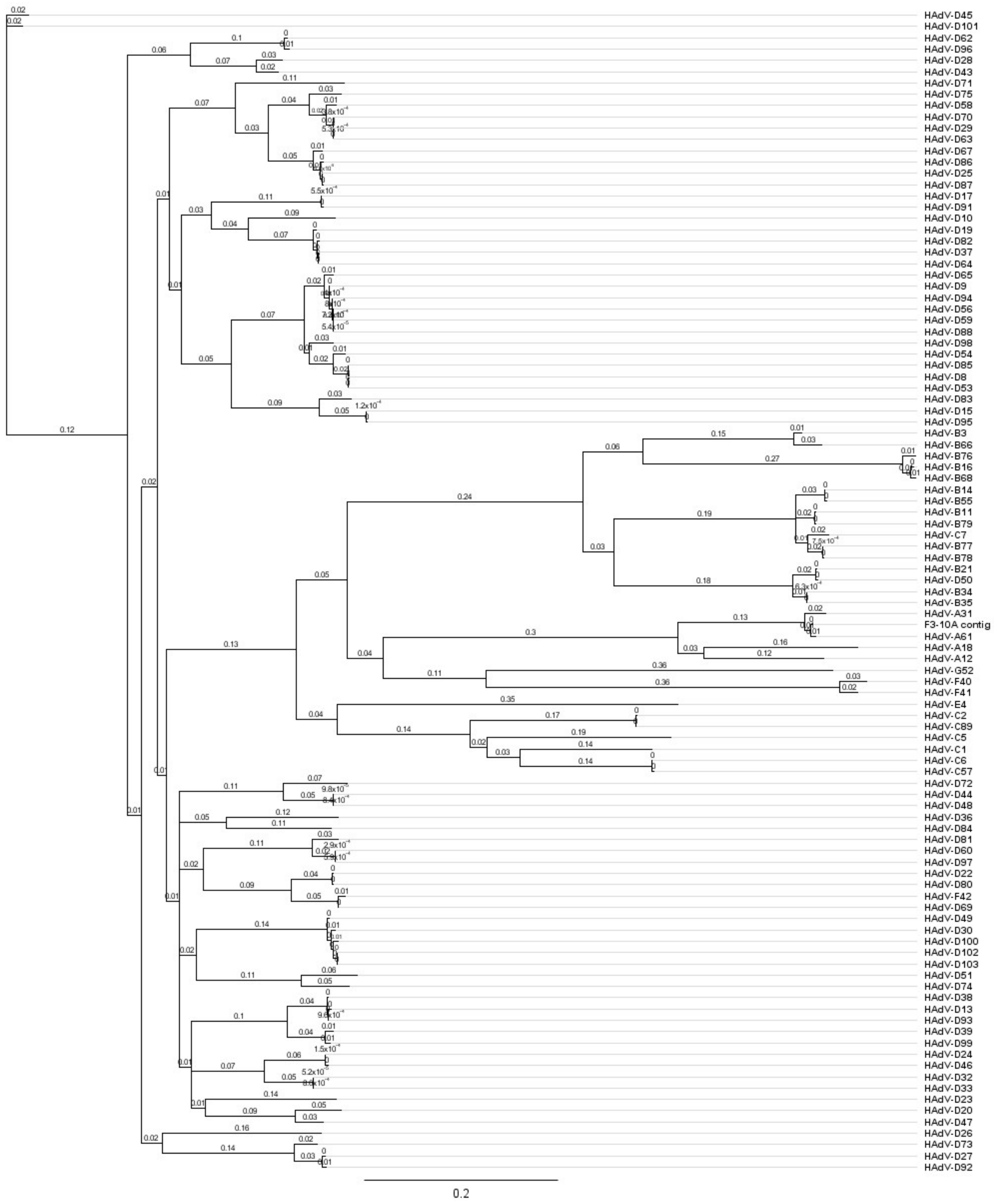
HAdV-D45
HAdV-D101
HAdV-D62
HAdV-D96
HAdV-D28
HAdV-D43
HAdV-D71
HAdV-D75
HAdV-D58
HAdV-D70
HAdV-D29
HAdV-D63
HAdV-D67
HAdV-D86
HAdV-D25
HAdV-D87
HAdV-D17
HAdV-D91
HAdV-D10
HAdV-D19
HAdV-D82
HAdV-D37
HAdV-D64
HAdV-D65
HAdV-D9
HAdV-D94
HAdV-D56
HAdV-D59
HAdV-D88
HAdV-D98
HAdV-D54
HAdV-D85
HAdV-D8
HAdV-D53
HAdV-D83
HAdV-D15
HAdV-D95
HAdV-B3
HAdV-B66
HAdV-B76
HAdV-B16
HAdV-B68
HAdV-B14
HAdV-B55
HAdV-B11
HAdV-B79
HAdV-C7
HAdV-B77
HAdV-B78
HAdV-B21
HAdV-D50
HAdV-B34
HAdV-B35
HAdV-A31
F3-10A contig
HAdV-A61
HAdV-A18
HAdV-A12
HAdV-G52
HAdV-F40
HAdV-F41
HAdV-E4
HAdV-C2
HAdV-C89
HAdV-C5
HAdV-C1
HAdV-C6
HAdV-C57
HAdV-D72
HAdV-D44
HAdV-D48
HAdV-D36
HAdV-D84
HAdV-D81
HAdV-D60
HAdV-D97
HAdV-D22
HAdV-D80
HAdV-F42
HAdV-D69
HAdV-D49
HAdV-D30
HAdV-D100
HAdV-D102
HAdV-D103
HAdV-D51
HAdV-D74
HAdV-D38
HAdV-D13
HAdV-D93
HAdV-D39
HAdV-D99
HAdV-D24
HAdV-D46
HAdV-D32
HAdV-D33
HAdV-D23
HAdV-D20
HAdV-D47
HAdV-D26
HAdV-D73
HAdV-D27
HAdV-D92
0.2

Supplementary material 6. Characterization of enterovirus (a) and rhinovirus (b) contigs detected in the gastroenteritis pools using the enterovirus typing tool by RIVM (v.0.1).

(a)

Sample	Contig length	BLAST score	Genotype	VP1 Serotype, Sub-Genogroup	Classification
F1-3A	7,400	80.481865	CV-A10	CV-A10	Picornaviridae Enterovirus A
	7,193	83.12013	E-6	E-6	Picornaviridae Enterovirus B
	7,011	95.69187	CV-A6	CV-A6	Picornaviridae Enterovirus A
	2,696	83.11688	CV-B5	CV-B5	Picornaviridae Enterovirus B
	471	83.6518	-	-	Picornaviridae Enterovirus B
	1,248	86.3124	-	-	Picornaviridae Enterovirus B
F3-10	680	91.87592	-	-	Picornaviridae Enterovirus B
	817	83.190186	-	-	Picornaviridae Enterovirus B
	722	84.02778	E-6	E-6	Picornaviridae Enterovirus B
	965	81.12033	E-6	E-6	Picornaviridae Enterovirus B
	2,835	85.472496	-	-	Picornaviridae Enterovirus B
S<1A	7,213	83.90564	CV-B5	CV-B5	Picornaviridae Enterovirus B
S<1B	2,737	93.78882	E-13	E-13	Picornaviridae Enterovirus B
	2,068	92.30769			
	1,367	91.93899			
S1-3A	6,865	80.99679	E-9	E-9	Picornaviridae Enterovirus B
	7,085	85.63747	E-3	E-3	Picornaviridae Enterovirus B
	6,650	89.35338	CV-A6	CV-A6	Picornaviridae Enterovirus A
	6,902	80.17743	CV-A10	CV-A10	Picornaviridae Enterovirus A
	2,608	93.66603	E-13	E-13	Picornaviridae Enterovirus B
	365	90.95891			Picornaviridae Enterovirus B

	1,363				Picornaviridae Enterovirus B
S1-3B	7,158	95.96199	CV-A16	CV-A16 , B1a	Picornaviridae Enterovirus A
	6,284	78.27817	CV-B1	CV-B1	Picornaviridae Enterovirus B
	436	87.006966	CV-A4	CV-A4	Picornaviridae Enterovirus A
	458	86.18421	CV-A4		Picornaviridae Enterovirus A
	1,105	85.791855	CV-A4	CV-A4	Picornaviridae Enterovirus A
	2,122	83.577156	CV-A8		Picornaviridae Enterovirus A
	564	83.27402	EV-A71		
S1-3C	1,224	84.72222	E-9	E-9	Picornaviridae Enterovirus B
	635	86.456696			Picornaviridae Enterovirus B
	614	83.68679			Picornaviridae Enterovirus B
	794	86.901764			Picornaviridae Enterovirus B
S1-3B (unassigned)	1,454	82.09366	E-16	E-16	Picornaviridae Enterovirus B
	741	80.13514	E-16	E-16	Picornaviridae Enterovirus B
	358	92.20056	NTP	NTP	Picornaviridae Enterovirus B

(b)

Sample	Contig length	BLAST score	Genotype	VP1 Serotype, Sub-Genogroup	Classification
F3	597	90.60403	HRV-A60		Picornaviridae Rhinovirus A
	483	90.47619	HRV-A60		Picornaviridae Rhinovirus A
	592	88.983055	NTP		Picornaviridae Rhinovirus A
S1	459	94.77124	HRV-B52		Picornaviridae Rhinovirus B
	574	91.78322	HRV-B52		Picornaviridae Rhinovirus B
	1,155	92.03463	HRV-B52		Picornaviridae Rhinovirus B
	766	92.81985	HRV-B52		Picornaviridae Rhinovirus B
	626	93.29073	NTP		Picornaviridae Rhinovirus B
	413	91.26214	NTP		Picornaviridae Rhinovirus B
	451	93.11111	NTP		Picornaviridae Rhinovirus B
	409	93.887535	NTP		Picornaviridae Rhinovirus B
S2	6,499	86.74773	HRV-A78		Picornaviridae Rhinovirus A

Supplementary material 7. Characterization of non-enterovirus Picornaviridae contigs detected on the fecal pools analyzed, including parechovirus and cardiovirus.

Pool	Contig length	Classification	VP1 Genotype	% VP1 Identity	% VP1 Coverage	% Genome Identity	% Genome Coverage
S<1B	7,248	Parechovirus A	HPeV-1	87.8	100	87.8	98.5
S1-3B	796	Parechovirus A	-	-	-	95.6	10.8
	471	Parechovirus A	-	92.0	7.1	95.8	6.4
	1,695	Parechovirus A	-	-	-	95.4	23.1
	343	Parechovirus A	-	-	-	95.0	4.7
	6,949	Parechovirus A	HPeV-6	95.4	100	95.9	94.6
S3-10	7,942	Cardiovirus B	SAFV-2	94.7	100	90.7	98.7

Supplementary material 8. Phylogenetic trees of retrieved parechovirus contigs. Trees were constructed by using tree view software within Geneious (software version 11.0 (https://www.geneious.com) maximum-likelihood (Tamura-Nei model, Bootstrap of 1,000 replicates) analysis. Phylogenetic trees were constructed using the (a), full length of the VP1 genomic sequence (b), full human parechovirus complete genomes. The labels on the branch indicate the number of substitutions per site.

a.

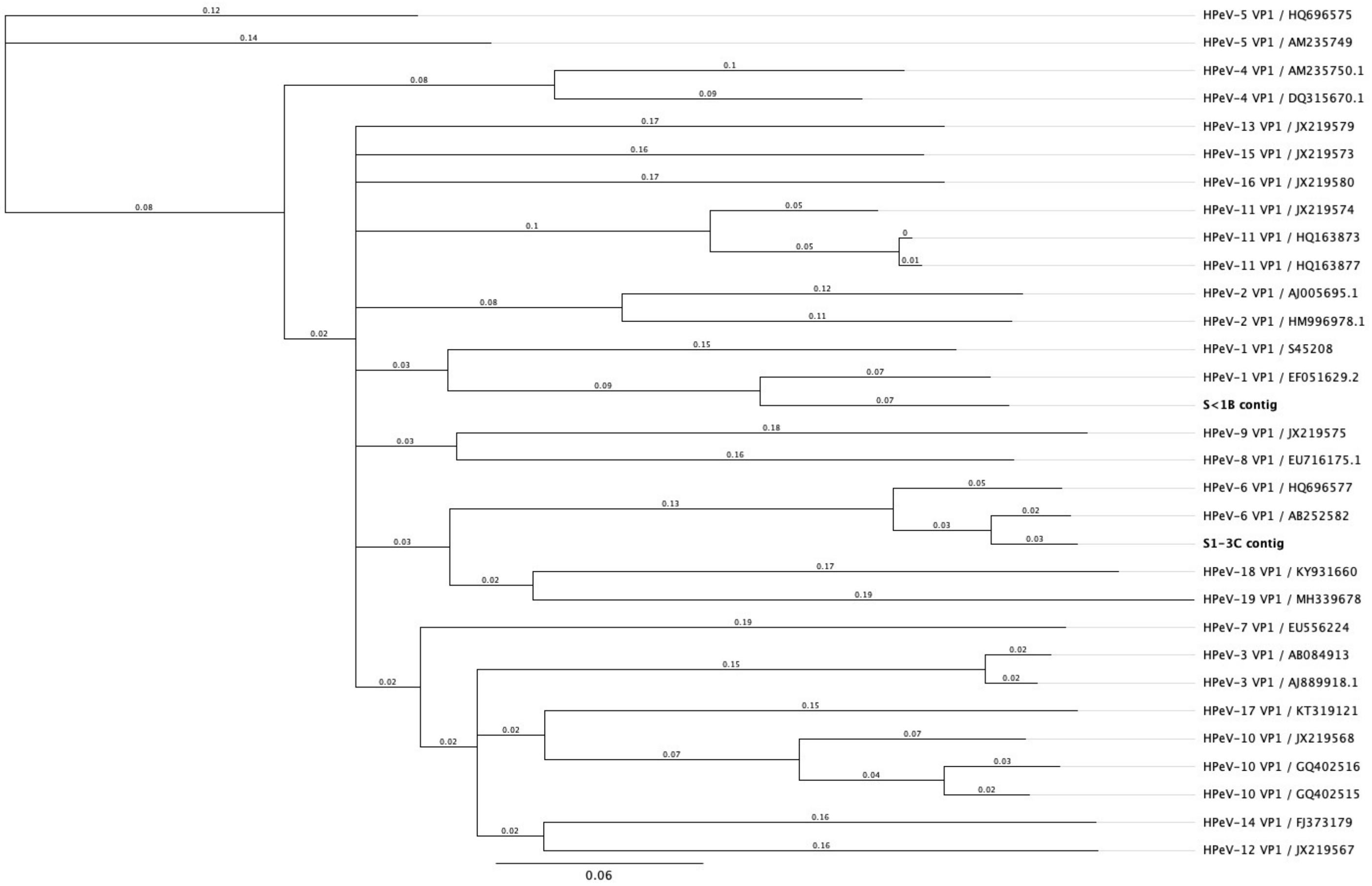

b.

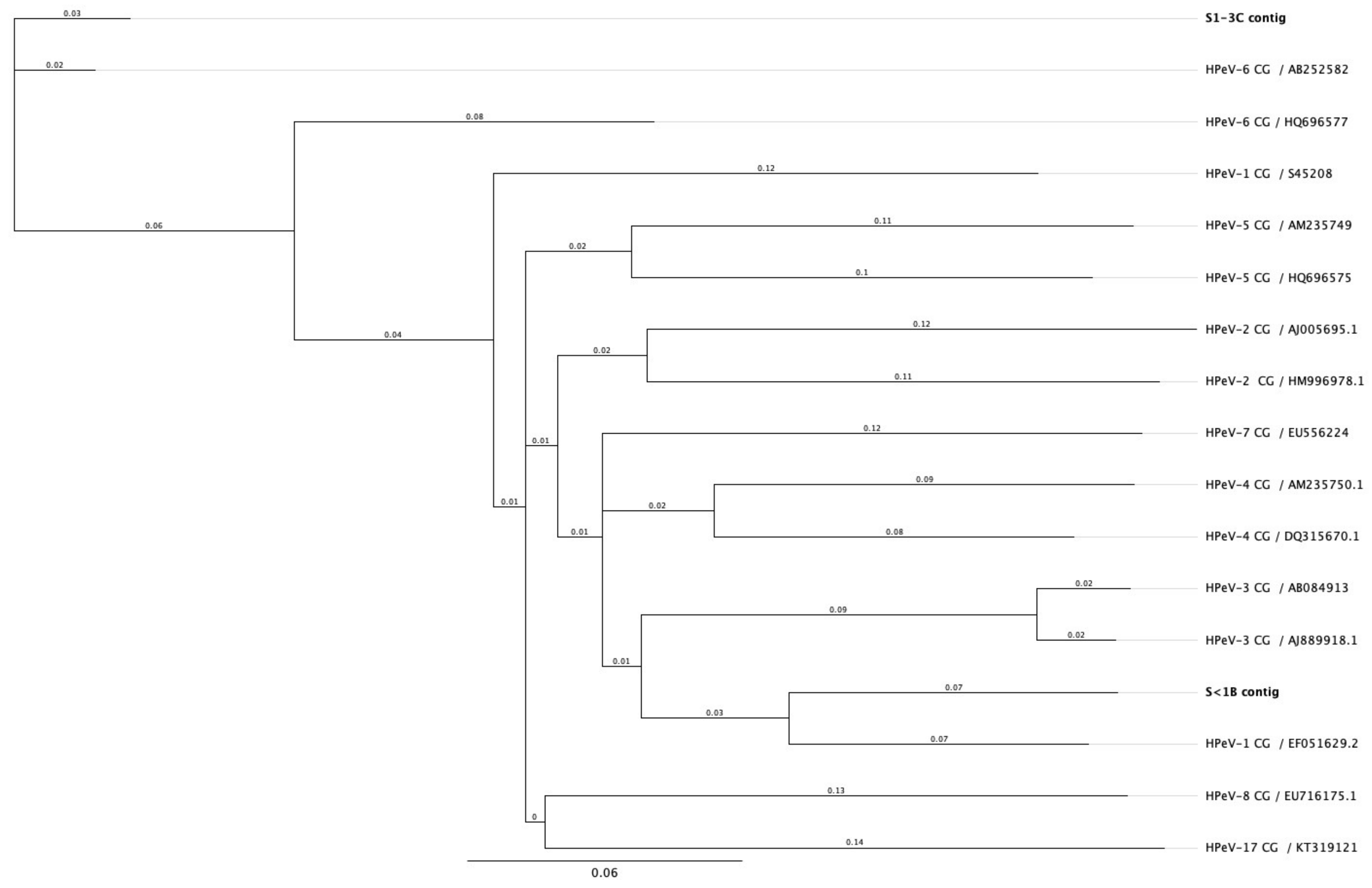
S1-3C contig
HPeV-6 CG / AB252582
HPeV-6 CG / HQ696577
HPeV-1 CG / S45208
HPeV-5 CG / AM235749
HPeV-5 CG / HQ696575
HPeV-2 CG / AJ005695.1
HPeV-2 CG / HM996978.1
HPeV-7 CG / EU556224
HPeV-4 CG / AM235750.1
HPeV-4 CG / DQ315670.1
HPeV-3 CG / AB084913
HPeV-3 CG / AJ889918.1
S<1B contig
HPeV-1 CG / EF051629.2
HPeV-8 CG / EU716175.1
HPeV-17 CG / KT319121
0.06
